# Drugs Associated with Adverse Effects in Vulnerable Groups of Patients

**DOI:** 10.3390/clinpract14030080

**Published:** 2024-05-31

**Authors:** Claudia Simona Ștefan, Aurel Nechita, Oana-Maria Dragostin, Ana Fulga, Elena-Lăcrămioara Lisă, Rodica Vatcu, Ionut Dragostin, Cristian Velicescu, Iuliu Fulga

**Affiliations:** 1 Research Centre in the Medical-Pharmaceutical Field, Faculty of Medicine and Pharmacy, “Dunarea de Jos” University of Galati, 35 AL Cuza st, 800010 Galati, Romania; claudia.stefan@ugal.ro (C.S.Ș.); ana.fulgaa@gmail.com (A.F.); elena.lisa@ugal.ro (E.-L.L.); rodica.vatcu@gmail.com (R.V.); ionut.dragostin@yahoo.com (I.D.); fulgaiuliu@yahoo.com (I.F.); 2 Faculty of Medicine, “Grigore T Popa” University of Medicine and Pharmacy Iasi, 16 University Street, 700115 Iaşi, Romania; cristianvelicescu@yahoo.com

**Keywords:** toxicity, diuretics, fluoroquinolones, children, pregnant women, elderly people

## Abstract

In recent years, a series of recommendations have been issued regarding the administration of drugs because of awareness of the serious side effects associated with certain classes of drugs, especially in vulnerable patients. Taking into account the obligation of the continuous improvement of professionals in the medical fields and the fact that we are in the midst of a “malpractice accusations pandemic”, through this work, we propose to carry out a “radiography” of the scientific literature regarding adverse effects that may occur as a result of the interaction of drugs with the physiopathological particularities of patients. The literature reports various cases regarding different classes of drugs administration associated with adverse effects in the elderly people, such as fluoroquinolones, which can cause torsade de pointes or tendinopathy, or diuretics, which can cause hypokalemia followed by torsade de pointes and cardiorespiratory arrest. Also, children are more prone to the development of adverse reactions due to their physiological particularities, while for pregnant women, some drugs can interfere with the normal development of the fetus, and for psychiatric patients, the use of neuroleptics can cause agranulocytosis. Considering the physiopathological particularities of each patient, the drug doses must be adjusted or even completely removed from the treatment scheme, thus requiring the mandatory active participation both of clinician pharmacists and specialists in the activity of medical-pharmaceutical analysis laboratories within the structure of hospitals.

## 1. Introduction

As a result of the awareness of the serious side effects associated with certain classes of drugs that are administered to vulnerable categories of patients, recommendations regarding cautious administration of drugs have begun to be more and more frequent. 

Thus, in the case of elderly patients, there are three main causes that can lead to an increase in the concentration of drugs in the plasma and tissues, increasing the risk of overdose: the decrease in muscle mass, the reduction of the total amount of water, and the increase in adipose tissue. For water-soluble drugs, the volume of distribution decreases, thus increasing their blood concentration, while for fat-soluble drugs, accumulation in the fatty tissue increases the duration of action.

In addition, it is well known that as people get older, there is an increase in the consumption of prescription drugs, and if food supplements are included in the treatment scheme, these prevalence rates increase substantially. The correct drug treatment of elderly patients becomes a challenge for several reasons: first, they use more drugs than any other age group, increasing both the risk of adverse effects and drug interactions, thus determining a more difficult compliance. Secondly, elderly persons are more likely to have chronic disorders that can be aggravated by certain classes of drugs or that can affect the response to drugs. An example in this respect is the absorption of the active substance or its elimination, while aging can modify the pharmacodynamics and pharmacokinetics of drugs [1].

In the case of pregnant women, the increase in total body water volume leads to an increase in plasma volume. Thus, the volume of distribution for hydrophilic drugs increases, as their plasma concentrations decrease [2]. The interaction of drugs with the human body becomes even more sensitive when it comes to pregnant women because not only is the future mother exposed to adverse reactions but the fetus is too. Thus, the impact of drugs on fetal organogenesis is crucial and can have serious consequences [3]. Gastrointestinal tract tonus, acidity, and motility are reduced during pregnancy, and this could interfere with drug absorption or excretion, and ultimately, drug metabolism may be affected at certain stages of pregnancy. In addition, during pregnancy, the pharmacokinetics and pharmacodynamics are altered, the total blood volume increases by 30–40% (1500–1800 mL), and the extravascular volume increases during the second and third trimester, resulting in a reduced plasma concentration of different drugs [4,5].

In the case of newborns, the oral absorption of fat-soluble substances is influenced by the reduced activity of gastrointestinal enzymes. The insufficient development of biotransformation enzymes in newborns, compared to adults, requires careful adjustment of doses [6]. The drug class with the most risks involved in children is that of aminoglycosides [7]. 

The treatment of psychiatric patients being long-term, the Food and Drug Administration (FDA) of the United States of America warns against prescribing antipsychotics due to the increased risk of cardiovascular mortality resulting from chronic use [8,9,10]. In addition, antipsychotics are commonly associated with weight gain, impaired glycemia, glucose tolerance, disturbances in lipid metabolism, and clinical progression to the metabolic syndrome, also increasing the risk of diabetes mellitus, which may be attributable to both weight gain and intrinsic effects on glucose metabolism [11].

The high toxicity of antineoplastic drugs and their low therapeutic index, together with the health status of cancer patients, are aspects that place them in the risk category. Almost everyone who takes an antipsychotic experiences some degree of side effects. The particularities of these patients have been analyzed in the literature; for example, Peccatori and colleagues show that cardiac toxicity and secondary leukemia can occur following treatments with anthracyclines and alkylating agents [12]. 

Hence, considering all these particularities, a careful and continuous monitoring is necessary, whether we are talking about elderly patients, pregnant women, children, or oncological or psychiatric patients, in order to prevent the occurrence of interactions between their physio-pathological factors and different classes of medicines. 

## 2. Materials and Methods

For our study, we have used multiple combinations of keywords, such as toxicity, diuretics, fluoroquinolones, angiotensin converting enzyme inhibitors (ACEI), children, pregnant women, and elderly people. Initially, the abstracts of the articles were analyzed using the following inclusion criteria (vulnerable categories of patients, adverse reactions to common drugs), eliminating those scientific works that did not contain information of interest for our study. The studies were organized according to the established objectives, emphasizing the most important adverse reactions, supported by real cases reported so far in the literature, as follows: information about the vulnerable categories, followed by the presentation of real cases in different pharmacological classes: antimicrobial chemotherapy; antiarrhythmics; cardiotonic glycosides; antihypertensives; antitumor drugs; drugs with action on the central nervous system.

## 3. Results. Case Studies in Different Pharmacological Classes 

### 3.1. Antimicrobial Chemotherapy 

#### 3.1.1. Fluoroquinolones 

**Figure 1:**
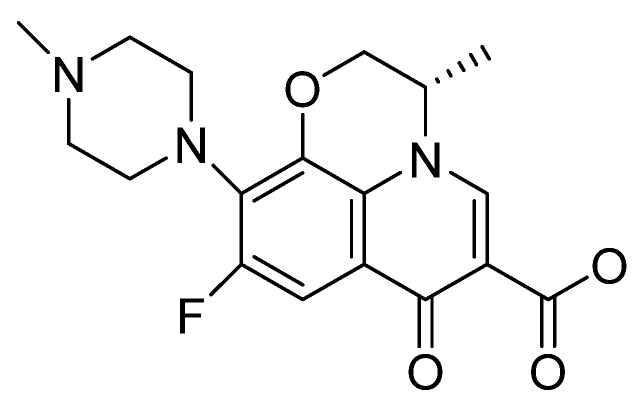
Chemical structure of the fluoroquinolone levofloxacin.

Fluoroquinolones (Figure 1), referred to as “new quinolones”, are a family of broad-spectrum antibiotics that are frequently used in the treatment of a wide range of infections, being represented by ciprofloxacin, norfloxacin, levofloxacin, ofloxacin, lomefloxacin, and fleroxacin.

They are relatively safe and well tolerated, but at the same time, they are responsible for numerous adverse reactions, especially in elderly patients, inducing polymorphic ventricular tachycardia—torsade de pointes or tendinopathy. Also, phototoxicity is one of the frequent adverse reactions, being correlated with substitution at the X-8 position in the aromatic nucleus—Figure 1 [13,14,15].

The most common symptom of tendinopathy associated with fluoroquinolones is pain, usually of sudden onset. Other symptoms include swelling, tenderness, erythema, or itching in tendon areas and functional disability. The most common tendon affected is the Achilles tendon, but others can also be affected [16,17,18,19]. An example reported for the use of fluoroquinolones in vulnerable categories is that of a 91-year-old patient, for whom the doctor prescribed levofloxacin, 500 mg, once a day, for 10 days, for a possible upper respiratory tract infection. On the seventh day of treatment, suddenly, the patient presented instability and impaired walking, so the neurologist referred him to a rehabilitation center. After examining the legs, a halo sign was observed in both heels. The Thompson Test was performed, being positive in both legs, which confirmed that the heel fracture was preceded by levofloxacin treatment [20].

Another case of tendon rupture was reported for a 70-year-old man patient with pain in both heels and difficulty in walking. In this case, the patient experienced bilateral spontaneous, non-traumatic ruptures of the Achilles tendon following treatment with ciprofloxacin [21].

#### 3.1.2. Aminoglycosides 

Aminoglycosides are natural antibiotics or semi-synthesis products with an aminocyclitol-aminoglycoside structure which show structural similarities with some polysaccharides in the capsule and the wall of bacterial cells, the mechanism of action being bactericidal. They are a low-therapeutic-index antibiotic, with a therapeutically effective plasma concentration close to the toxic one. Thus, it is well known that the main side effects of aminoglycosides are oto- and nephrotoxicity, and while ototoxicity is manifested by cochlear and vestibular damage, nephrotoxicity occurs due to the accumulation of aminoglycosides in the renal parenchyma [22]. A clinical study demonstrating the ototoxicity of amikacin was conducted at a regional hospital in Cape Town, South Africa. This was carried out on 94 children (45 boys and 49 girls) who were audiometrically tested, their average age being 43 months. A total of 30 of the children had already proven to be infected with *Mycobacterium tuberculosis* upon admission to the hospital. For 52 of them, the presence of the bacterium was confirmed in the hospital. Due to the disadvantaged area when the study was conducted, 28 children were HIV-positive, 20 of whom had already started retroviral therapy. The remaining 12 children were treated with rifampicin.

Also, the following audiometric testing have pointed out that 23 children lost their hearing; 44 children developed hearing impairment and 27 children remained with normal hearing abilities. Both hearing impairment and hearing loss have been shown to be common in children treated for multidrug-resistant tuberculosis with amikacin [7].

Regarding the nephrotoxicity of aminoglycosides, a study published in 2023 analyzes which aminoglycoside antibiotic affects kidney cells the most. The study was conducted on six antibiotics of this class: gentamicin, tobramycin, amikacin, neomycin, plazomicin, and streptomycin. To provide an overview of the renal damage that aminoglycosides cause, both acute and chronic nephrotoxicity were examined. Acute nephrotoxicity is expressed by an increase in plasma creatinine concentration of more than 0.5 to 1 mg/dL (44 to 88 micromol/L) or a 50% increase in plasma creatinine from baseline, which occurs in from 10 to 20% of patients. Also, according to these data, aminoglycoside-induced acute kidney injury may occur in up to 20 to 33% of children receiving the treatment, while gentamicin was found to be one of the most dangerous aminoglycosides that cause severe kidney damage. The use of an alternative is a better option to avoid renal toxicity, and if gentamicin is necessary for the patient, it should be administered under supervision and with special care [23].

### 3.2. Antiarrhythmics 

Amiodarone is considered an antiarrhythmic agent class III, prolonging the third phase antiarrhythmic agent, the repolarization phase in which there is normally a low permeability to calcium and an increased permeability to potassium [24,25]. Its metabolite, desethylamiodarone, has a long half-life (over 28 days) and a lipophilic nature that causes it to accumulate in tissues in high concentrations and interfere with phospholipid metabolism [26]. In addition, this can sometimes cause serious adverse reactions in all organs [27,28]. Thus, the two iodine atoms in the central benzene ring, which give it a structure similar to triiodothyronine, are responsible for thyroid gland disorders and hypo- and hyperthyroidism [29] by inhibiting iodine transport into the thyroid gland [30]. 

Pulmonary toxicity is less common than thyroid and eye damage but is the most dangerous event for the patient, leading to respiratory insufficiency or even death, and can occur in both acute and subacute/chronic forms [31]. Among the first events, we can expect bronchospasm exacerbation, bronchial asthma, interstitial pneumonia, diffuse alveolar hemorrhage; among secondary events, we can expect lipoid pneumonia, chronic eosinophilic pneumonia, nodules, or pulmonary masses [32,33]. A case of subacute pulmonary toxicity was reported by Sahah M., and collaborators, 2023, in an 81-year-old woman who arrived at the hospital with dyspnea that had started a week before. Due to suspicion of pulmonary toxicity induced by amiodarone, treatment with this antiarrhythmic was discontinued and systemic corticosteroid therapy was initiated. After 3–4 days of corticosteroid treatment, the symptoms showed significant improvement [31]. Another case of amiodarone-induced pulmonary toxicity, but this time of an acute type, was presented by Rodriguez-Rivera A.A. et al. in 2023, involving a 61-year-old patient with chronic obstructive pulmonary disease (COPD) and heart failure. Acute pulmonary toxicity induced by amiodarone was suspected, and therapy with this antiarrhythmic was discontinued. Also in this case, treatment with corticosteroids was initiated, significantly improving symptoms and radiographic results [34]. 

### 3.3. Cardiotonic Glycosides 

**Figure 2:**
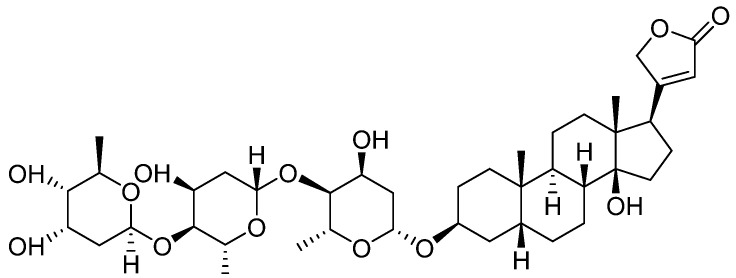
Chemical structure of Digoxin (12 beta-Hydroxydigitoxin).

Digitoxin is a cardiotonic glycoside extracted from the plant *Digitalis lanata* [35], and by its hydroxylation, 12 beta-hydroxy digitoxin, digoxin—Figure 2—is obtained with better hydrophilicity and an increase in *Vd* in skeletal muscles, heart, and kidneys. However, in the elderly, *V*d decreases with the reduction of the skeletal muscles, which causes an increase in the plasma concentration; this is the reason why it is important to monitor digoxin plasma levels, especially for this category of patients [36]. 

A case of digoxin toxicity, leading to the occurrence of a rare tachycardia known as bidirectional ventricular tachycardia, was recently presented by Nair A., et al. in 2023 [37]. The case involves a 32-year-old patient diagnosed with mitral valve insufficiency, moderate left ventricular dysfunction, and atrial fibrillation. For the past two months, the patient had been undergoing treatment with furosemide, amiodarone, and digoxin. Serologically, the patient exhibited hypokalemia with serum potassium levels of 2.5 mmol/L, likely due to furosemide administration and high serum digoxin levels. Treatment was initiated to correct hypokalemia through intravenous potassium chloride and magnesium sulfate administration, as severe hypokalemia coexisted with hypomagnesemia. After three days of therapy, serum digoxin levels returned to normal [37].

To all that have been presented are added the aggravating factors associated with digitalis poisoning, such as serum concentration of digoxin, advanced age, renal dysfunction, coronary artery disease, pulmonary damage, diabetes, hypo/hyperthyroidism, metabolic factors (hypokalemia, hypomagnesemia, hypoxemia, hypernatremia, hypercalcemia), hydro-electrolyte disturbances, and history of heart failure [38,39,40]. 

Furthermore, the decreasing use of digoxin over the last two decades, the improvement of the technology for detecting its levels in the blood, and a better knowledge of drug interactions have led to a considerable decrease in the incidence of digitalis poisoning.

### 3.4. Antihypertensives 

The compounds in this class of antihypertensive drugs are contraindicated during pregnancy, causing teratogenicity and even fetal death. Their use in the second and third trimesters of pregnancy was associated with a characteristic fetopathy including renal dysplasia, pulmonary hypoplasia, oligohydramnios, and limb contractures. 

#### 3.4.1. Angiotensin-Converting Enzyme Inhibitors

The use of angiotensin-converting enzyme inhibitors (ACE inhibitors) represents a major step forward in the treatment of arterial hypertension [41]. In general, adverse effects associated with the use of these ACE inhibitors have included hypotension, hyperkalemia, or irritating nonproductive cough, while rash and angioneurotic edema are allergic symptoms. In addition, fetal hypotension caused by angiotensin-converting enzyme inhibitors (ACEIs) is the cause of fetal anuria and oligohydramnios, with the consequence being the occurrence of teratogenic effects [5]. For this class of drugs, the literature presents the case of a 28-year-old woman in her second pregnancy, with hypertension treated with ramipril (an ACE inhibitor) every day, which caused the birth of a 880 g baby girl with an increasing respiratory distress [42].

#### 3.4.2. Angiotensin II Receptor Blockers 

Angiotensin II receptor blockers have similar effects to angiotensin-converting enzyme inhibitors but with a different mechanism of action [43,44]. Their use during the second or third trimester of pregnancy can have a serious effect on the fetus, being associated with the impairment of fetal renal function which can cause a decrease in amniotic fluid volume, neonatal renal failure, anuria, fetal hypotension, and fetal or neonatal death [45,46]. A reported case in the literature is that of a 31-year-old woman with hypertension, treated with Irbesartan 2 × 150 mg/day, who presented to the gynecologist at 8 weeks after her last menstrual period, and Irbesartan treatment was immediately discontinued. In this case, the abnormality found during the physical examination after born was polydactyly, with an extra finger on the right hand and another toe on the left foot [47]. 

### 3.5. Anti-Tumor Drugs 

#### 3.5.1. Bleomycin

Bleomycin is an effective antitumor agent for the treatment of lymphoma, testicular tumors, and squamous cell carcinomas. The adverse reactions of bleomycin include neutropenia, genotoxicity, hyperpigmentation, and pulmonary toxicity [48], which includes pulmonary edema, diffuse alveolar damage, and chronic pneumonitis with fibrosis and hypersensitivity reactions, while pulmonary toxicity can sometimes be rapidly fatal [49]. A case of bleomycin toxicity was presented by Hu S. and Palmer L.B in 2023 [50], involving a 68-year-old patient who was treated for stage 3 left testicular tumor with chemotherapy including bleomycin, carboplatin, and etoposide over four treatment cycles. Two weeks after completing the treatment, he developed breathing difficulties and non-productive cough. 

Due to suspicion of bleomycin toxicity, systemic corticosteroid treatment and antibiotic therapy were initiated, but following multidisciplinary consultations, the patient is undergoing evaluation for lung transplantation [50]. A similar case of severe pulmonary damage caused by bleomycin toxicity is that of a 45-year-old man diagnosed with mixed testicular cancer. Radiological examination showed opacification in both lung lobes and the presence of a pneumothorax, while bronchoscopy revealed airway edema with hyperemia. Also in this case, pulmonary toxicity of bleomycin was suspected, which is why high-dose corticosteroid therapy and oxygen therapy were initiated [51]. 

#### 3.5.2. Cisplatin

In the case of Cisplatin, widely used to treat osteosarcoma, hepatoblastoma, neuroblastoma, germ cell tumors, head and neck cancers, and some central nervous system tumors, multiple toxicities have been reported, including peripheral neuropathy, nephrotoxicity, nausea/vomiting, and ototoxicity. Once in the kidney, cisplatin undergoes biotransformation to cysteinyl glycine conjugates and other higher thiols that are believed to cause toxicity; also, a higher cisplatin dose can result in hepatotoxicity. This may be caused by the oxidative stress which results from a reduction in glutathione [52,53,54]. One of the major complications of cisplatin chemotherapy is cerebral toxicity, with studies showing apoptosis of neuronal cells induced by cisplatin administration, a consequence of the induction of pro-apoptotic factors, granule cell proliferation, and Purkinje cell migration [55], while in cases of children, they turned out to be more susceptible to ototoxicity [56,57]. 

### 3.6. Drugs with Action on the Central Nervous System

#### 3.6.1. Hypnotic—Barbiturates

**Figure 3:**
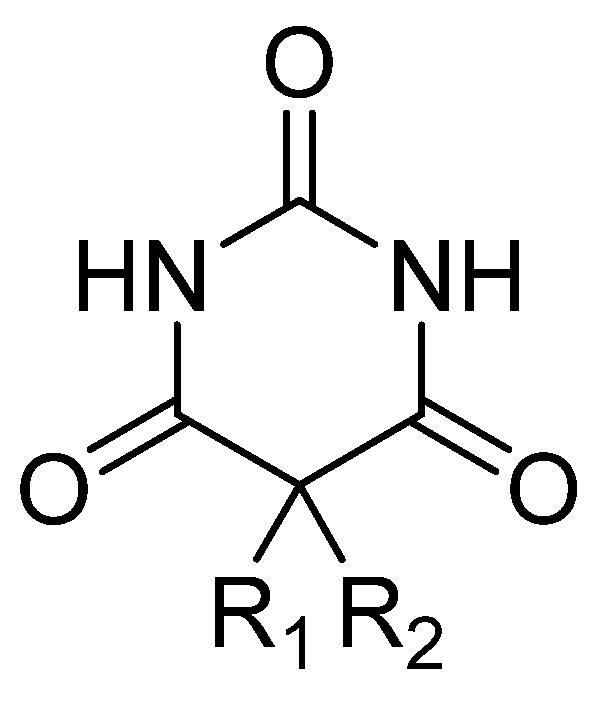
General chemical structure of barbiturate derivatives.

Barbiturates (Figure 3) have various onsets of action, durations, half-lives, and toxic levels, depending on the lipid solubility and rate of metabolic inactivation. The onset of action for oral administration ranges from 20 to 60 min, and intravenous administration can range from almost immediate to 5 min. The high lipid solubility of some barbitures (amobarbital, pentobarbital, and thiopental) allow them to be well absorbed and rapidly redistributed [58]. 

Barbiturates are derivatives of barbituric acid (2,4,6 triketohexahydropyrimidine—Figure 3): substitute for C5 in the case of hypnotic barbiturates; N-alkylates and thiobarbituric acid derivatives for narcotic barbiturates. The hypnotic action is increased by asymmetric substituents and hypnophoric groups such as branched substituents, double and triple bonds, and halogens. Depending on the size of the lipid–water partition coefficient, diffusion in the CNS, and redistribution, narcotic barbiturates (especially thiobarbituric compounds and N-derivatives) have high lipid solubility and, as a result, ultrashort latency and duration. Barbituric acid itself does not possess central depressant activity; substitutions, primarily at position 5, confer sedative hypnotic properties. Replacement of the oxygen atom in the 2-position with a sulfur atom, producing a thiobarbiturate, confers a greater degree of lipid solubility compared to the oxybarbiturates. In general, the more lipid-soluble the barbiturate, the faster the onset; therefore, there is a shorter duration and a greater degree of hypnosis [59].

In this class, the case of a 45-year-old man who was brought to the emergency room after being found in a deep coma is presented in the literature [60]. He had a positive history of epilepsy for 27 years and a history of head trauma at 40 years without serious consequences. His relatives found an empty bottle of phenobarbital pills with him, and therefore a suicide attempt was suspected. Toxicology screening revealed the phenobarbital level to be up to 180 mg/mL while the valproate and phenytoin levels were 10 and 1.7 mg/mL. In the present case, worsening seizures may be induced by phenobarbital toxicity; thus, an electroencephalographic evaluation was essential to manage the manifestations appropriately.

#### 3.6.2. Antipsychotics

The benefits of antipsychotic drugs are sometimes hidden by their adverse effects. These effects range from relatively minor tolerability (e.g., mild sedation or dry mouth) to very unpleasant side-effects (e.g., constipation, akathisia, sexual dysfunction), to painful (e.g., acute dystonia), to disfiguring side-effects (e.g., weight gain, tardive dyskinesia), and to life-threatening side-effects (e.g., myocarditis, agranulocytosis) [61,62]. Since the treatment of psychiatric patients is long-term, the Food and Drug Administration (FDA) of the United States warns against prescribing antipsychotics due to the increased risk of cardiovascular mortality resulting from chronic use [9,11].

The atypical antipsychotics are well absorbed from the gastrointestinal tract but undergo a significant first-pass metabolism. They are highly lipophilic, protein bound, and accumulate in the brain, lung, and other tissues, having a large volume of distribution. However, both typical and atypical antipsychotics have side effects that exist along a spectrum and are related to the unique receptor-binding profiles of each agent [63,64,65].

Antipsychotic overdose produces a gamut of manifestations that affect multiple organ systems, the most serious toxicity involving the cardiovascular system and the central nervous system (CNS). All typical and atypical antipsychotics cause sedation due to CNS histamine H_1_ receptor blockade in therapeutic dosing and is pronounced in overdose. Sedation is most prominent with clozapine and quetiapine [63]. The most common cardiovascular effects that occur after atypical antipsychotic overdose are tachycardia, mild hypotension, and prolongation of the QT interval [66]. 

At the cardiovascular level, clozapine most commonly causes myocarditis and cardiomyopathy. In this regard, Shnoda M., et al. presented, in 2023 [65], a case of acute clozapine toxicity in a 47-year-old man diagnosed with schizophrenia, who arrived at the emergency department with persistent tachycardia. Cardiac magnetic resonance imaging (MRI) and modified Lake-Louise criteria led to the diagnosis of clozapine-induced myocarditis, and it was withdrawn from therapy, being replaced with risperidone [65].

#### 3.6.3. Antidepressants 

Symptoms of an antidepressant venlafaxine overdose may include tachycardia, unusual drowsiness, dilated pupils, seizures, vomiting, cardiac arrhythmias, low blood pressure, muscle aches or pains, or dizziness. Stopping venlafaxine suddenly can lead to serious side effects such as irritability, tiredness, restlessness, anxiety, insomnia, sleep disturbances, nightmares, headaches, sweating, dizziness, tingling or “pins and needles”, tremors, confusion, nausea, vomiting, or diarrhea [67]. In addition, severe toxicity of venlafaxine, especially when combined with other antidepressants such as SSRIs, SNRIs, or MAOIs, can lead to serotonin syndrome, which can present with a spectrum of clinical findings, including autonomic hyperactivity, mental status changes, and neuromuscular abnormalities [68].

For this class of medicines, the literature reports the case of a 29-year-old woman with major depressive disorder who suddenly developed serotonin syndrome during low-dose venlafaxine monotherapy (37.5 mg/day) with symptoms of restlessness, tremors, diarrhea, vomiting, ataxia, tachycardia, and myoclonus. The patient recovered within 2 h after receiving emergency prochlorperazine and lorazepam, while venlafaxine was discontinued and she was discharged home. This example draws attention to the risk of serotonin syndrome when the patient receives not only a combination of two antidepressants but also a single potent serotonergic agent such as venlafaxine [69].

## 4. Conclusions

Considering the physio-pathological particularities of each patient, it is necessary to adjust the drug doses, replace them, or eliminate them from the treatment plan. Pharmacovigilance requires special attention in the approach of any therapy to vulnerable patient groups, such as the elderly people, pregnant women, children, or oncological patients. To have a better perspective on the medication among these patients, all the variables that could lead to a possible unwanted outcome must be included. All the particularities that interfere with the action of the drug, variables related to pharmacodynamics, pharmacokinetics, polymedication, drug interactions, physiological changes, and different pathologies, require the adjustment of each therapy. Therefore, there are two main approaches to optimizing drug therapy in the vulnerable groups of patients: the first one is represented by using appropriate drugs according to medical indications, and the second one consists of avoiding the adverse effects of drugs by ensuring the correct dosage, stopping unnecessary drugs, and avoiding drug–drug and drug–disease interactions.

Also, the drug classes that involve risk among vulnerable patient categories, such as fluoroquinolones, aminoglycosides, angiotensin-converting enzyme inhibitors, and cardiotonic glycosides, require a very careful evaluation of their introduction into therapy and the analysis of their benefit. Additionally, with their use in therapy, compliance and the evolution of therapeutic effectiveness, as well as the patient’s condition, are rigorously monitored.

All this supports the need for the presence of a clinical pharmacist in the multidisciplinary team to bring additional information related to the pharmacokinetics, pharmacodynamics, and pharmacotoxicology of pharmaceutical substances, the creation of a therapeutic scheme free of drug interactions, the inclusion in therapy of adjuvant substances that support the needs of a recovering body (antioxidants in cancer diseases, in cardiovascular pathologies and dyslipidemias, the use of pre- and probiotics in antibiotic therapy, supplementing the necessary vitamins and mineral substances in women during maternity and children during growth), and the development of information programs for women of childbearing age regarding the negative influence of certain classes of drugs on the conception and development of the fetus, supported by doctors and the mass media.

Equally, it is necessary to establish toxicology departments within all clinical laboratories and the presence of a pharmacist specialized in medical-pharmaceutical laboratory analyses to ensure early detection of the plasma levels reached by pharmaceutical substances (in therapeutic or toxic areas).

## Data Availability

No new data were created.

## References

[1] Qato D.M., Wilder J., Schumm L.P., Gillet V., Alexander G.C. (2016). Changes in prescription and over-the-counter medication and dietary supplement use among older adults in the United States, 2005 vs. 2011. JAMA Intern. Med..

[2] Brookfield K.F., Su F., Elkomy M.H., Drover D.R., Lyell D.J., Carvalho B. (2016). Pharmacokinetics and placental transfer of magnesium sulfate in pregnant women. Am. J. Obstet. Gynecol..

[3] Pack A.M., Davis A.R., Kritzer J., Yoon A., Camus A. (2009). Antiepileptic drugs: Are women aware of interactions with oral contraceptives and potential teratogenicity. Epilepsy Behav..

[4] Duncombe D., Wertheim E.H., Skouteris H., Paxton S.J., Kelly L. (2008). How well do women adapt to changes in their body size and shape across the course of pregnancy. J. Health Psychol..

[5] Halpern D.G., Weinberg C.R., Pinnelas R., Mehta-Lee S., Economy K.E., Valente A.M. (2019). Use of Medication for Cardiovascular Disease During Pregnancy: JACC State-of-the-Art Review. J. Am. Coll. Cardiol..

[6] Aranda J.V., Beharry J.V. (2020). Pharmacokinetics, pharmacodynamics and metabolism of caffeine in newborns. Semin. Fetal Neonatal Med..

[7] Seddon J.A., Thee S., Jacobs K., Ebrahim A., Hesseling A.C., Schaaf H.S. (2013). Hearing loss in children treated for multidrugresistant tuberculosis. J. Infect..

[8] Oh J., Nam H., Park S., Chae J.-H., Kim T.-S. (2021). Decreased cardiovascular death in schizophrenia patients treated with antipsychotics: A Korean national cohort study. Schizophr. Res..

[9] Wang P.S., Schneeweiss S., Avorn J., Fischer M.A., Mogun H., Solomon D.H., Brookhart M.A. (2005). Risk of Death in Elderly Users of Conventional vs. Atypical Antipsychotic Medications. N. Engl. J. Med..

[10] Berger S.J., Hofer A. (2023). Sicherheitsaspekte bei der Behandlung mit Clozapin. Neuropsychiatrie.

[11] Zito M.F., Marder S.R. (2020). Rethinking the risks and benefits of long-term maintenance in schizophrenia. Schizophr. Res..

[12] Peccatori P.A., Azim H.A. (2009). Pregnancy in breast cancer survivors: A need for proper counseling. Breast.

[13] Yabe K., Goto K., Jindo T., Sekiguchi M., Furuhama K. (2005). Structure–phototoxicity relationship in Balb/c mice treated with fluoroquinolone derivatives, followed by ultraviolet-A irradiation. Toxicol. Lett..

[14] Davis A.E., Kennelley G.E., Amaye-Obu T., Jowdy P.F., Ghadersohi S., Nasir-Moin M., Paragh G., Berman H.A., Huss W.J. (2024). The phenomenon of phototoxicity and long-term risks of commonly prescribed and structurally diverse drugs. J. Photochem. Photobiol..

[15] Eljaaly K., Alkhalaf A., Alhifany A.A., Alshibani M. (2020). Photosensitivity induced by lomefloxacin versus other fluoroquinolones: A meta-analysis. J. Infect. Chemother..

[16] Van der Linden P.D., Sturkenboom M.C.J.M., Herings R.M.C., Leufkens H.G.M., Stricker B.C. (2002). Fluoroquinolones and risk of Achilles tendon disorders: Case-control study. Br. Med. J..

[17] Khaliq Y., Zhanel G.G. (2003). Fluoroquinolone-associated tendinopathy: A critical review of the literature. Clin. Infect. Dis..

[18] Baik S., Lau J., Huser V., McDonald C.J. (2020). Association between tendon ruptures and use of fluoroquinolone, and other oral antibiotics: A 10-year retrospective study of 1 million US senior Medicare beneficiaries. BMJ.

[19] Shu Y., Zhang Q., He X., Liu Y., Wu P., Chen L. (2022). Fluoroquinolone-associated suspected tendonitis and tendon rupture: A pharmacovigilance analysis from 2016 to 2021 based on the FAERS database. Pharmacoepidemiology.

[20] Fernández-Cuadros M.E., Casique-Bocanegra L.O., Albaladejo-Florín M.J., Gómez-Dueñas S., Ramos-Gonzalez C., Pérez-Moro O.S. (2019). Bilateral Levofloxacin-Induced Achilles Tendon Rupture: An Uncommon Case Report and Review of the Literature. Clin. Med. Insights Arthritis Musculoskelet. Disord..

[21] Kawtharani F., Masrouha K.Z., Afeiche N. (2016). Bilateral Achilles Tendon Ruptures Associated with Ciprofloxacin Use in the Setting of Minimal Change Disease: Case Report and Review of the Literature. J. Foot Ankle Surg..

[22] Manen S., Bost-Bru C., Wroblewski I., De Crescenzo M., Mortamet G. (2021). Aminoglycoside prescription: Compliance with national guidelines in a pediatric hospital. Arch. Pédiatrie.

[23] Pandey V.K., Shivani S., Patel A., Shivani, Kaur M. (2023). Aminoglycoside induced kidney injury and its pharmacological treatment–overview. J. Popul. Ther. Clin. Pharmacol..

[24] Beaulieu-Jones B.R., Lin B., Phillips A.M., Haime M., Quin J.A. (2023). Postoperative Atrial Fibrillation After Surgical Aortic Valve Replacement: Amiodarone and Warfarin Use. J. Surg. Res..

[25] Maraffa J.M. (2024). Amiodarona, Encyclopedia of Toxicology.

[26] Pérez-Ruiz T., Martínez-Lozano C., García-Martínez M.D. (2008). Simultaneous determination of amiodarone and its metabolite desethylamiodarone by high-performance liquid chromatography with chemiluminescent detection. Anal. Chim. Acta.

[27] Erven L., Schalij M.J. (2010). Amiodarone: An effective antiarrhythmic drug with unusual side effects. Educ. Heart Clin. Pharmacol..

[28] Papiris S.A., Triantafillidou C., Kolilekas L., Markoulaki D., Manali E.D. (2010). Amiodarone: Review of pulmonary effects and toxicity. Drug Saf..

[29] Siddiqui M.A., Khan A., Zaka M. (2016). A Review of Structure Activity Relationship of Amiodarone and Its Derivatives. Open J. Med. Chem..

[30] Ganesan K., Bradley B., Jones D.W., Solomon S. (2021). A Case Report on Type 2 Amiodarone Induced Thyrotoxicosis and Hypercalcemia. Am. J. Med. Sci..

[31] Shah M., Rogers L., Tribuls K., Sorresso D.J. (2023). Acute amiodarone-induced pulmonary toxicity. Chest.

[32] Camus P., Fanton A., Bonniaud P., Camus C., Foucher P. (2004). Interstitial lung disease induced by drugs and radiation. Respiration.

[33] Terzo F., Ricci A., D’Ascanio M., Raffa S., Mariotta S. (2019). Amiodarone-induced pulmonary toxicity with an excellent response to treatment: A case report. Respir. Med. Case Rep..

[34] Rodriguez-Rivera A.A., Melendez D.A.C., Sultan S., Goyal A., Sharma B. (2023). Amiodarone-induced pulmonary toxicity leading to acute respiratory distress syndrome. Chest.

[35] Bhusare B.P., John C.K., Bhatt V.P., Nikam T.D. (2018). In vitro propagation of Digitalis lanata Ehrh. through direct shoot regeneration—A source of cardiotonic glycoside. Ind. Crops Prod..

[36] Ziff O.J., Kotecha D. (2016). Digoxin: The good and the bad. Trends Cardiovasc. Med..

[37] Nair A., Abhiraj R., Aggarwal P. (2023). Digoxin toxicity and a rare tachycardia. Vis. J. Emerg. Med..

[38] Luciano K.S., Bogo V.S., Schulze M.L., Ronsoni R.d.M. (2021). Bidirectional Ventricular Tachycardia Due to Digitalis Intoxication. J. Card. Arrythmias.

[39] Pita-Fernández S., Lombardía-Cortiña M., Orozco-Veltran D., Gil-Guillén V. (2011). Clinical manifestations of elderly patients with digitalis intoxication in the emergency department. Arch. Gerontol. Geriatr..

[40] Doherty J.E. (2010). The Clinical Pharmacology of Digitalis Glycosides: A Review. Am. J. Med. Sci..

[41] Atalay E., Özdemir M.T., Çiğsar G., Omurca F., Aslan N., Yildiz M., Gey Z.B. (2015). Angiotensin Converting Enzyme Inhibitor-related Angioedema: A Case of an Unexpected Death. Iran. J. Allergy Asthma Immunol..

[42] Shrim A., Berger H., Kingdom J., Hamoudi A., Shah P.S., Koren G. (2005). Prolonged exposure to angiotensin-converting enzyme inhibitors during pregnancy. Can. Fam. Physician.

[43] Villanacci V., Del Sordo R. (2021). Angiotensin II receptor antagonist (Olmesartan) associated gastro-entero-colopathy. The multiform expressions of damage due to this class of drugs. Dig. Liver Dis..

[44] Shibata S., Fujita T. (2024). 22-Renin Angiotensin Aldosterone System Blockers, Hypertension.

[45] Velázquez-Armenta E.Y., Han J.Y., Choi J.S., Yang K.M., Nava-Ocampo A.A. (2007). Angiotensin II receptor blockers in pregnancy: A case report and systematic review of the literature. Hypertens. Pregnancy.

[46] Clark C.R., Khalil R.A. (2024). Regulation of vascular angiotensin II type 1 and type 2 receptor and angiotensin-(1–7)/MasR signaling in normal and hypertensive pregnancy. Biochem. Pharmacol..

[47] Gersak K., Cvijic M., Cerar L.K. (2009). Angiotensin II receptor blockers in pregnancy: A report of five cases. Reprod. Toxicol..

[48] Sani S.M.S., Sahranavard M., Yazdanabad M.J., Shamsi M.S., Elyasi S., Mohammadpour A.H., Sathyapalan T., Arasteh O., Ghavami V., Sahebkar A. (2022). The effect of concomitant use of Colony-Stimulating factors on bleomycin pulmonary toxicity–A systematic review and meta-analysis. Int. Immunopharmacol..

[49] Keijzer A., Kuenen B. (2007). Fatal pulmonary toxicity in testis cancer with bleomycin-containing chemotherapy. J. Clin. Oncol..

[50] Hu S., Palmer L.B. (2023). Unilateral pulmonary fibrosis in setting of bleomycin toxicity and left hemidiaphragm paralysis. Chest.

[51] Arif S., Jafri F., Tahir O. (2020). Spontaneous pneumomediastinum and multiple pneumothoraces: An unusual presentation of bleomycin-related lung toxicity. Chest.

[52] Aldossary S.A. (2019). Review on Pharmacology of Cisplatin: Clinical use, toxicity and mechanism of resistance of cisplatin. Biomed. -Pharmacol. J..

[53] Quintanilha J.C.F., Saavedra K.F., Visacri M.B., Moriel P., Salazar L.A. (2019). Role of epigenetic mechanisms in cisplatin-induced toxicity. Crit. Rev. Oncol./Hematol..

[54] Cardinaal R.M., Huizing E.H., Veldman J.E., Smoorenburg G.F. (2000). Cisplatin-induced ototoxicity: Morphological evidence of spontaneous outer hair cell recovery in albino guinea pigs?. Hear. Res..

[55] Yoo K.H., Tang J.J., Rashid M.A., Cho C.H., Corujo-Ramirez A., Choi J., Bae M.G., Brogren D., Hawse J.R., Hou X. (2021). Nicotinamide Mononucleotide Prevents Cisplatin-Induced Cognitive Impairments. Found. Cancer J. Driv. Transform. Sci..

[56] Moke D.J., Luo C., Millstein J., Knight K.R., Rassekh S.R., Brooks B., Orgel E. (2021). Prevalence and risk factors for cisplatin-induced hearing loss in children, adolescents, and young adults: A multi-institutional North American cohort study. Lancet Child Adolesc. Health.

[57] Truong M.T., Winzelberg J., Chang K.W. (2007). Recovery from cisplatin-induced ototoxicity: A case report and review. Int. J. Pediatr. Otorhinolaryngol..

[58] Suddock J.T., Cain M.D. (2022). Barbiturate Toxicity. StatPearls.

[59] Van den Hondel K.E. (2020). The rise of suicides using a deadly dose of barbiturates in Amsterdam and Rotterdam, the Netherlands, between 2006 and 2017. J. Forensic Leg. Med..

[60] Hassanian-Moghaddam H., Ghadiri F., Shojaei M., Zamani N. (2016). Phenobarbital overdose presenting with status epilepticus: A case report. Seizure.

[61] Murphy A., Bentur H., Dolan C., Bugembe T., Gill A., Appleton R. (2014). Outpatient anti-epileptic drug prescribing errors in a Children’s Hospital: An audit and literature review. Seizure.

[62] Koenig M., McCollum B., Spivey J.K., Coleman J.K., Shelton C.H.A.R.L.E.S., Cotes R.O., De Leon J.O.S.E. (2022). Four cases of myocarditis in US hospitals possibly associated with impaired metabolism of clozapine and a comparison with previously published cases. Neuropsychopharmacol. Hung..

[63] DuBois D. (2005). Toxicology and overdose of atypical antipsychotic medications in children: Does new necessarily mean safer?. Curr. Opin. Pediatr..

[64] Kumra S., Oberstar J.V., Sikich L., Findling R.L., McClellan J.M., Vinogradov S., Schulz S.C. (2008). Efficacy and tolerability of second-generation antipsychotics in children and adolescents with schizophrenia. Schizophr. Bull..

[65] Shnoda M., Sagalov A., Patel H., Siddique M., Hegde S. (2023). Clozapine-induced myocarditis: A rare case of myocarditis with life-threatening implications. J. Am. Coll. Cardiol..

[66] Tan H.H., Hoppe J., Heard K. (2009). A systematic review of cardiovascular effects following atypical antipsychotic overdose. Am. J. Emerg. Med..

[67] Saad M.A., El-Sahar A.E., Sayed R.H., Elbaz E.M., Helmy H.S., Senousy M.A. (2019). Venlafaxine Mitigates Depressive-Like Behavior in Ovariectomized Rats by Activating the EPO/EPOR/JAK2 Signaling Pathway and Increasing the Serum Estradiol Level. Neurotherapeutics.

[68] Singh D., Saadabadi A. (2022). Venlafaxine. StatPearls.

[69] Pan J.J., Shen W.W. (2003). Serotonin Syndrome Induced by Low-Dose Venlafaxine. Ann. Pharmacother..

